# Understanding Freshness Perception from the Cognitive Mechanisms of Flavor: The Case of Beverages

**DOI:** 10.3389/fpsyg.2017.02360

**Published:** 2018-01-11

**Authors:** Jérémy Roque, Malika Auvray, Jérémie Lafraire

**Affiliations:** ^1^Centre de Recherche Pernod Ricard, Créteil, France; ^2^Center for Food and Hospitality Research, Institut Paul Bocuse, Écully, France; ^3^Institute of Intelligent Systems and Robotics, Université Pierre et Marie Curie, Paris, France; ^4^Institut Jean Nicod, Ecole Normale Supérieure, Paris, France

**Keywords:** freshness, flavor, multisensory perception, crossmodal correspondences, beverages

## Abstract

Freshness perception has received recent consideration in the field of consumer science mainly because of its hedonic dimension, which is assumed to influence consumers’ preference and behavior. However, most studies have considered freshness as a multisensory attribute of food and beverage products without investigating the cognitive mechanisms at hand. In the present review, we endorse a slightly different perspective on freshness. We focus on (i) the multisensory integration processes that underpin freshness perception, and (ii) the top–down factors that influence the explicit attribution of freshness to a product by consumers. To do so, we exploit the recent literature on the cognitive underpinnings of flavor perception as a heuristic to better characterize the mechanisms of freshness perception in the particular case of beverages. We argue that the lack of consideration of particular instances of flavor, such as freshness, has resulted in a lack of consensus about the content and structure of different types of flavor representations. We then enrich these theoretical analyses, with a review of the cognitive mechanisms of flavor perception: from multisensory integration processes to the influence of top–down factors (e.g., attentional and semantic). We conclude that similarly to flavor, freshness perception is characterized by hybrid content, both perceptual and semantic, but that *freshness* has a higher-degree of specificity than *flavor*. In particular, contrary to flavor, freshness is characterized by specific functions (e.g., alleviation of oropharyngeal symptoms) and likely differs from flavor with respect to the weighting of each sensory contributor, as well as to its subjective location. Finally, we provide a comprehensive model of the cognitive mechanisms that underlie freshness perception. This model paves the way for further empirical research on particular instances of flavor, and will enable advances in the field of food and beverage cognition.

## Introduction

The perceived freshness of food and beverages has been generally considered as the result of several sensory contributions: olfactory, gustatory, tactile, trigeminal, visual, and auditory (Westerink and Kozlov, 2003; Labbe et al., 2009a,b; Saint-Eve et al., 2010). Databases—Google Scholar and Science Direct—were searched over all years of records using pre-specified terms (freshness, refreshing, perception, consumers) in the consumer science literature. Our searches identified more than one hundred studies dealing with freshness, with a large fraction of the studies focusing on freshness of fruits and vegetables (see Péneau, 2005 for a review) and other food products like bread and biscuits (e.g., Heenan et al., 2008). However, most have considered freshness as a multisensory attribute of food and beverage products without investigating the multisensory integration processes and cognitive mechanisms^1^at hand. Furthermore, the consumer science literature clearly embeds two distinct types of freshness whose cognitive underpinnings are probably very different. To better characterize the subject of the present review, it is thus necessary to first address the sematic ambiguity of the term “freshness”.

The semantic ambiguity of “freshness” is straightforward. When experiencing products containing both liquid and solid components as well as several tastants and odorants (e.g., cocktails), consumers can characterize them as fresh based on either the overall multisensory experience (involving for instance coldness, sourness, or a menthol odor), or on the aging of the organic ingredients it contains (e.g., the age of the mint leaves in a mojito). For instance, Péneau (2005, p. 6) defined freshness of fruits and vegetables as the “level of closeness to the original product, in terms of distance, time and treatment”. Thus, it seems that at least two meanings may be conveyed by the word “freshness”. In this review, we will focus on the first meaning. We will also focus on the multisensory integration processes at the perceptual level and on the top–down factors that may influence those processes. We will consider the case of beverages, by reviewing the perceptual and cognitive mechanisms that cause a particular beverage to be perceived as fresh.

According to Labbe et al. (2009a), the concepts of refreshing and freshness (in the first sense mentioned above) seem closely linked because they have common sensory drivers such as coldness and mint flavor. The notion of refreshment in beverages was also shown to strongly correlate with a thirst-quenching sensation (Labbe et al., 2009b). In the present review, we address the concept of *freshness* instead of *refreshing* because the term refreshing is more directed toward the post-consumption phase, together with the impact fresh beverages have on arousal or thirst-quenching sensation (Labbe et al., 2009a). In the following sections, we will thus refer to “perceived freshness” because it covers both the expected and the actual sensation of a given product.

Freshness, in that restricted sense, has been analyzed at various levels of description. From the *physiological* level of analysis, freshness perception seems linked to the alleviation of unpleasant physical symptoms, such as elevated body temperatures or mouth dryness, during the consumption of a cool or fresh beverage (see Labbe et al., 2009a for a review). With respect to the *sensory* level, several sensory descriptors have been shown to positively or negatively influence freshness perception. Some of them have also been associated with the term “refreshing”, as well as with psychophysiological factors involved in freshness perception: “thirst-quenching” and “mouth-wetting” (Labbe et al., 2009a,b, 2011). According to consumers, coldness and sourness are the common sensory properties that enhance freshness perception in food and beverages (Zellner and Durlach, 2002), waters (Labbe et al., 2011), and soft drinks (McEwan and Colwill, 1996; Fenko et al., 2009; Saint-Eve et al., 2010; Zhang et al., 2016). On the other hand, sweetness (McEwan and Colwill, 1996; Guinard et al., 1998; Labbe et al., 2009b) and thickness (Scriven et al., 1989; McEwan and Colwill, 1996; Guinard et al., 1998; Labbe et al., 2009b; McCrickerd et al., 2015) were associated with decreased freshness perception. Regarding colors, a clear color was found to enhance thirst-quenching and refreshing perceptions of soft drinks (Clydesdale et al., 1992; Zellner and Durlach, 2003). Red and orange colors were also associated with an increase in thirst-quenching perception of fruit-based drinks (Zellner and Durlach, 2002). According to different studies, smells like mint, orange (Zellner and Durlach, 2002; Labbe et al., 2009b), peppermint, lemon (Fenko et al., 2009), citrus, and peach (Martin et al., 2005) were judged to be the most refreshing aromas for food and beverages, whereas chocolate was most commonly listed as the least refreshing (Zellner and Durlach, 2002; Labbe et al., 2009b). Other kinds of perceptual features such as carbonation in beverages have also been shown to positively influence freshness perception (McEwan and Colwill, 1996).

However, to go beyond simply listing the perceptual features that contribute to freshness perception, it is necessary to identify the mechanisms that integrate them into a unified freshness perception. The binding of multisensory inputs into one unified percept has been investigated and extensively discussed in the case of flavor (see Auvray and Spence, 2008 for a review). So why does the literature lack of information on the mechanisms of freshness perception?

One possible, simple explanation of this gap is that it would be redundant with the literature on flavor. We will show that this answer should be qualified: even if some redundancies will be unavoidable, freshness differs from flavor in some important respects. Indeed, flavor is more generic than freshness, which in turn seems more generic than particular flavors (e.g., mint flavor). All freshness instances are systematically confounded with particular flavors (e.g., mint or citrus flavors) but all flavors are not necessarily confounded with perceived freshness. This type of relationship corresponds to what is called a “taxonomic hierarchy” between categories or concepts (Murphy, 2002, p. 325). Specifying at which level of the hierarchy freshness is located (superordinate, basic-level, or subordinate) would lead us beyond the scope of the present review. Rather, we aim to determine what the higher degree of specificity of freshness implies, regarding the multisensory mechanisms at hand. Furthermore, we will argue that thoroughly considering a particular instance of flavor such as freshness may shed light on some theoretical debates on flavor perception.

First of all, competing theses regarding the representational nature of flavor and freshness will be reviewed. It will be discussed whether flavor and freshness have to be considered as cases of synesthetic experiences, object representations, or rather as perceptual categories (see Section “The Representational Nature of Flavor and Freshness”). This conceptual clarification will allow us to move to the central question of the present review which is the characterization of the perceptual and cognitive mechanisms that underlie the experience of freshness. The existing literature on the cognitive underpinnings of flavor perception will be used as a heuristic to determine whether or not the same cognitive underpinnings apply *mutatis mutandis* to the perception of freshness. We will focus on the cases of crossmodal interactions and correspondences, which contribute to the experiences of flavor and likely to freshness (see Section “Crossmodal Interactions and Correspondences”). Then, the memory and attentional aspects impacting the perceptual and cognitive processes mentioned above will be described (see Sections “Memory, Expectations, and Knowledge” and “Attention”). Finally, the particular conditions that influence the degree of integration of a multisensory perception, such as congruency and the unity assumption (see Section “Particular Conditions that Influence the Degree of Integration”), will be reviewed. This will allow us to build a preliminary model of freshness perception and provide specific empirical research hypotheses about the putative crossmodal interactions and correspondences that influence the experience of freshness.

## The Representational Nature of Flavor and Freshness

In this section, we will consider both the computational processes underpinning flavor and freshness perception and the types of representations for which flavor and freshness are eligible. The processes will be addressed first by introducing a distinction between multisensory integration and sensory fusion. Then, the representational nature of flavor will be questioned by considering various types of representations: synesthetic experiences, object representations, and perceptual categories. Each time, the representational nature (structure and content) of freshness will be compared to that of flavor.

### Sensory Fusion

During the consumption of a food or beverage, multiple sensory inputs are, under certain circumstances (see Section “Particular Conditions that Influence the Degree of Integration”), integrated by the central nervous system to potentially give rise to a unified flavor perception (e.g., Auvray and Spence, 2008). One of the main functions of the cognitive system is to resolve certain conflicts that occur during the integration process. Sometimes, the conflict resolution between two or more perceptual features can be such that each sensory contributor, or at least one of them, will lose its individual sensory characteristics. This phenomenon has been named sensory fusion (Verhagen and Engelen, 2006). Sensory fusion corresponds to a higher degree of sensory integration and to a particular level of unification between sensory features that can either belong to the same sensory channel (intramodal case) or to two distinct sensory modalities (crossmodal case). An intramodal instance of fusion occurs for instance when two colors (e.g., red and yellow) combine and consequently lose their initial individual sensory qualities to form a new sensation (i.e., red and yellow are merged into orange). The fusion process is specific to particular dimensions of the sensory signal, as it does not seem to occur between basic tastes for instance (e.g., McBurney and Gent, 1979). Indeed, sweetness and bitterness cannot be merged into a third basic taste. A crossmodal instance of fusion occurs when two or more sensory features belonging to distinct sensory modalities are combined and result in a distinct percept. In this case, at least one of the initial sensory inputs loses its respective modal identity (i.e., the perceptual format that specifies the type of sensory modality at the origin of the representation) and the fusion of the two forms one perceptual unit (see Verhagen and Engelen, 2006; Auvray and Spence, 2008 for reviews). For instance, it is generally assumed that a conflict occurs systematically between the spatial information conveyed by the taste signal and the spatial information conveyed by the smell signal, especially the retronasal one (Small and Prescott, 2005). The perceptual consequence of the conflict resolution by the cognitive system is that sensations originating in the nose are “referred to” or experienced as if they were transduced by the receptors in the mouth (Spence et al., 2014). Different terms have been used to describe this subjective phenomenon resulting from a sensory fusion between taste and smell such as the “location binding” (Stevenson et al., 1995), the “olfactory illusion” (Prescott, 1999), or the “mouth capture” (Shepherd, 2012). It is worth noticing that the strong claim that flavor perception is systematically underpinned by sensory fusion conflicts with the idea that humans still have the capacity to analyze a flavor into its parts (e.g., Stevenson, 2014).

In the case of freshness, it is likely that the modal identity of each sensory contributor is not always systematically accessible to the subject. Two non-exclusive hypotheses can be formulated to explain this phenomenon: i) the modal identity of the sensory contributor is not accessible because of a fusion occurring at some point during the multisensory integration and therefore the modal identity of certain contributors is lost, and/or ii) the modal identity of some contributors to freshness is intrinsically ambiguous. In fact, it has been shown that coldness can enhance the perception of freshness (Labbe et al., 2009a) and coldness is mediated by specific receptors found in trigeminal cold-sensing neurons. However, these receptors are widely distributed (on the tongue, in the nasal cavity, and in the peripheral nervous system) and can be activated either by cold temperatures or by different organic compounds with cooling properties such as menthone and menthol (for a review of mechanisms of temperature perception, see Patapoutian et al., 2003). Moreover, it has been shown that some people can experience the “thermal-taste illusion” in which a particular temperature experienced on the tongue will induce other taste sensations (Bajec and Pickering, 2008). Thus, in a nutshell, the mere fact of not being conscious of all the distinct sensory contributors to freshness may be due to a fusion occurring between two or more sensory features during the multisensory integration step, or to ambiguous activations of certain receptors, or, as is likely to both.

### Synesthetic Experience

The reasons why some people are often unable to tease apart the relative contributions of smell and taste to flavor perception (Spence and Piqueras-Fiszman, 2014) have been discussed in the previous section. In this section, one step further will be taken to consider why certain odors can elicit changes in the perception of a particular tastant (e.g., Stevenson et al., 1999). For instance, the sensory fusion of taste and smell described earlier can induce a crossmodal enhancement under certain circumstances (congruency dependent, see Section “Congruency” as well as task dependent and attention dependent, see Section “Attention”). The sweetness enhancement that can result from the sensory fusion between a sweet taste and a strawberry odor has been widely studied in flavor perception. When these two perceptual features are experienced together, the strawberry flavor stored in memory will induce a sweetness enhancement: people will tend to perceive the sweetness as more intense for a product with the strawberry odor as compared to a control odorless stimulus with the same concentration of sweet taste^2^. The fact that this pairing can lead to perceptual changes of the taste (i.e., higher sweetness ratings) have led some researchers to argue that flavor perception could result from a synesthetic experience (Stevenson and Boakes, 2004; Verhagen and Engelen, 2006).

**FIGURE 1:**
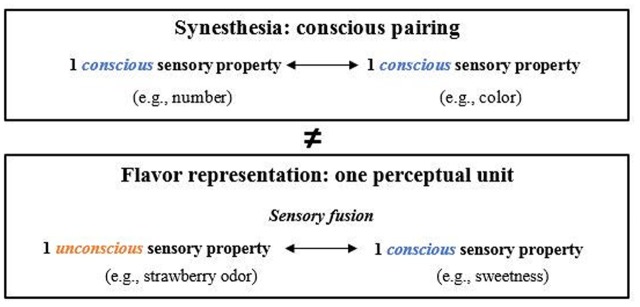
Differences between the cognitive processes underlying synesthesia and flavor representation. In both cases, there is spatio-temporal co-occurrence between two sensory properties belonging to two distinct modalities but they are not actually perceived at the same time in the case of flavor. In fact, only the result of the sensory fusion is perceived.

Synesthesia has been defined as a neurological condition in which the stimulation of one sensory or cognitive pathway leads to an automatic, involuntary experience in a second sensory or cognitive pathway (Cytowic, 2002). It can be characterized by four criteria that are jointly sufficient for individuating genuine forms of synesthesia: (a) the existence of a conscious pairing between an inducer (e.g., a number) and a concurrent (e.g., a color) perceived at the same time; (b) the relative idiosyncrasy of the pairing; (c) the automaticity of the process which causes an inevitable and involuntary experience of the concurrent when the inducer is present; and d) the consistency of the occurrence over time. Auvray and Farina (in press, see also Deroy and Spence, 2013) have suggested several arguments that question the appropriateness of considering flavor perception as the result of a synesthetic experience. The first criterion of a conscious pairing is not satisfied in the case of flavor perception. In fact, considering the sensory fusion occurring in flavor perception, it is the overall perceptual unit which is perceived by the participants and not the two individual sensory properties at the same time (see **Figure 1**). Regarding the second criterion, it has been shown that the multisensory processes underpinning flavor perception are experienced by everyone, at least intra-culturally (see section “Culture and Expertise”), thus it does not involve idiosyncrasy. Regarding the third criterion of automaticity of the process, controversial results have been found for flavor perception since the sensory fusion is dependent on the task and the attention of the participants (see Section “Attention”). Only the fourth criterion of consistency over time is verified regarding flavor perception. On the basis of the above-mentioned arguments, even if some arguments can be discussed, we argue that they are enough to consider the burden of proof to be on the proponents of the idea that flavor represents a genuine case of synesthetic experience. As freshness reasonably corresponds to a particular instance of flavor, it is then likely that the sensory contributors to freshness can also be bound in one perceptual unit. Moreover, people may often be unable to tease apart the relative contributions of each sensory contributor. Thus, similarly to flavor, we can reasonably argue that freshness does not result from a synesthetic experience.

### Object Representation and Categories

It has been put forward that “Object perception could represent a basic processing strategy applied to any sensory system that has to extract meaning from a complex stimulus array” (Stevenson, 2014, p. 1360). Hence, in order to further understand the mechanisms responsible for flavor perception, several authors have considered the object concept (e.g., Auvray and Spence, 2008; Small, 2012). Even though the object concept has been applied to vision, audition, and olfaction (see Stevenson, 2014), it has not been considered for other chemosensory modalities such as taste and chemesthesis (i.e., sensations due to chemical compounds). By listing the criteria that seem to characterize “object-hood” and making a distinction between the various sensory modalities that constitute flavor, Stevenson (2014) discussed whether flavors are integrated by default as objects (i.e. holistically), as a series of perceptual features, or both at different times. He described eight criteria for object-hood, taking into account the structural aspects of the stimulus and memory dependence that reflect respectively the bottom–up and the top–down processes contributing to the constitution of an object representation. One of the criteria that the notion of object-hood depends on is figure-ground segregation. The figure refers to the object itself and the background corresponds to the other perceptual features experienced at the same time and detected by the same receptor system. To illustrate that notion, let’s consider an example: when someone chews gum just before lunch time, the ingredients of the dish in the mouth will be experienced against a flavor background that consists of the remaining perceptual compounds of the gum. However, these persisting gum compounds will also impact the perception of the dish itself. According to Stevenson (2014), there is no evidence that a flavor object can be perfectly differentiated from its background, considering flavor as an object in the mouth. The figure-ground segregation criterion would not be satisfied.

Another reason that leads Stevenson to reject the idea that flavor is eligible for the status of object representation is that flavor-object-representations would have no additional function beyond that of olfactory or visual objects. Three potential functions are considered: detection, identification, and violation of expectancies. According to Stevenson (2009), foods are identified visually and via the orthonasal olfactory system before being placed in the mouth. Thus, forming flavor-object-representations in order to identify and detect foods would carry a useless cost for the cognitive system since these functions are achieved before the oral step. However, Stevenson acknowledges that the violation of expectancies hypothesis is more promising since flavor-object-representations could be compared to representations in memory. This comparison could then help the cognitive system to tease apart the expected properties from the unexpected properties of a given food or beverage. This comparison would then serve as a protective system since the perception of unexpected properties could help the individual to avoid ingesting poisonous substances. However, once again according to Stevenson this hypothesis is redundant since it is plausible that the violation of expectancies does not necessarily imply a comparison process between what is stored in memory and what is actually experienced.

Stevenson’s redundancy argument is clearly debatable since it is likely that the system based on taste is the precursor of a more fine grained protective system involving multiple sources of information conveyed by flavor objects. This is at least as plausible as Stevenson’s idea, from an evolutionary point of view. Furthermore, Stevenson’s argument seems quite inconsistent with the idea that flavor actually represents a functional sensory system with inputs from somatosensation, gustation, and olfaction where the meaning of the sensation would be more involved in the final representation than its precise organ or site of origin (Small and Prescott, 2005). This concept of flavor that focuses on the meaning of the sensation recalls Gibson’s (1966) ecological approach in which perceptual systems are defined by their *function*, rather than tied to the organ of transduction. Moreover, even if we concede that flavor objects have no obvious additional functions beyond those of gustatory, visual, or olfactory objects, this can be due to the generic character of the term flavor. Let us consider a more specific instance of flavor such as freshness. According to Labbe et al. (2009a), one possible function of freshness in the mouth is the alleviation of oropharyngeal symptoms (e.g., mouth dryness). Thus, by considering a particular instance of flavor such as freshness, it is possible to highlight specific functions of the flavor object experienced. Moreover, the different perceptual attributes and functions of a particular group of objects are generally contained in categories which have been defined by Rosch (1978) as groups of objects sharing common attributes that are most representative of items inside and less representative of items outside the corresponding category. Rosch (1978) has highlighted that one of the main functions of categories is to structure information and support recognition, discriminability, and inductive reasoning. Therefore, claiming that flavor does not have any specific function seems to imply that it is not eligible for the status of being a category. This is indeed what Stevenson’s analysis tends to suggest. According to Stevenson (2014), categorization is a *semantic process* which occurs mainly after the perceptual process (i.e., the construction of the object). However, we will see in the next paragraph how categorization has also been defined on the basis of perceptual processes.

A perceptual theory of knowledge was proposed by Barsalou (1999, p. 578) that highlights the convergence of cognition and perception. He described the nature of perceptual symbol systems as follows: “Subsets of perceptual states in sensory-motor systems are extracted and stored in long-term memory to function as symbols. As a result, the internal structure of these symbols is modal, and they are analogically related to the perceptual states that produced them.”. In other words, perceptual symbol systems represent schematic components of the multisensory experience and are intimately intertwined with higher order cognitive levels linked to semantics and memory, which help to structure information into categories. Thus, contrary to Stevenson’s idea that categorization is a semantic process that occurs after the perceptual process, according to Barsalou (1999) there is no real distinction between the structure of categories and the perceived features that belong to these categories.

To summarize, considering a specific instance of flavor such as freshness enables us to discuss the multisensory processes that can lead to its specific representational nature and more clearly characterize the perceptual and cognitive mechanisms at stake. For instance, the above-mentioned debate on categorization highlights the fact that a freshness representation could be considered as a category structured into a perceptual symbol system that consistently maintains the junction between the perceptual level and other higher levels of processing (e.g., semantic content). Thus, in order to further characterize the ordinary perceptual experience of freshness, it appears necessary to consider the interactions between the various perceptual features that contribute to freshness and other higher levels of processing. To do so, in the next section, the notion of crossmodal correspondences will be described and distinguished from the notion of crosslevel correspondences.

## Crossmodal Interactions and Correspondences

### The Crossmodal Perception of Flavor

Several crossmodal interactions have been reported in the case of flavor and the relative contributions of each sensory modality have already been partially documented (see Verhagen and Engelen, 2006; Shepherd, 2012, for reviews). A large body of research has also explored the evidence regarding the existence, and the consequences for human information processing, of a particular form of crossmodal interactions named *crossmodal correspondences*. Crossmodal correspondences have been defined as “the many nonarbitrary associations that appear to exist between different basic physical stimulus attributes, or features, in different sensory modalities” (see Spence, 2011, p. 972). The literature on flavor perception has highlighted the existence of crossmodal correspondences between many pairs of perceptual features belonging to different modalities constitutive of flavor such as tastes and sounds (Knöferle and Spence, 2012), auditory pitch and smell (Belkin et al., 1997), colors and odors/tastes (Spence et al., 2010, 2015), smells and shapes (Seo et al., 2010), and even shapes and tastes (Velasco et al., 2015).

The aim of this section is to go beyond the mere description of these correspondences in order to bring conceptual clarification. Indeed, crossmodal correspondence mechanisms refer to several phenomena occurring at distinct levels of information processing. Four principal types of crossmodal correspondences have been distinguished (see Spence, 2011 for a review): *Structural correspondences* are possibly innate, but may also depend on the maturation of neural structures for stimulus coding (e.g., between the loudness of a sound and the brightness of a light). *Statistical correspondences* are learned and result from the extraction of certain environmental regularities by the cognitive system. For instance, the correlation between the size, or mass, of an object and its resonant frequency (i.e., the larger the object, the lower the frequency). *Semantically mediated correspondences* may occur when common linguistic terms are used to describe two different stimuli. For instance, when a semantically ambiguous term refers to distinct sets of stimuli depending on the context, such as high and low, which can describe both pitch and elevation. *Emotionally mediated correspondences* consist of associations between basic perceptual features that seem to be mediated by certain dimensions of emotion such as the valence, or the level of arousal induced by the perceptual features.

On the basis of the above-mentioned typology of crossmodal correspondences, two fundamental types of correspondences appear: those occurring only at a perceptual level (e.g., between the size of an object and its resonant frequency), and those involving other levels of processing such as the semantically mediated correspondences and the emotionally mediated correspondences. In the next sections, the expression “crossmodal correspondences” will be used only to refer to the former. In other words, we will stick to the literal meaning of cross*modal* which implies that at least two sensory *modal*ities are involved and that the correspondence occurs at a perceptual level. By contrast, we will use “crosslevel correspondences” to refer to the associations involving another level of processing (e.g., linguistic or emotional).

Beyond the theoretical level, the existence of crossmodal correspondences can be inferred from their effects. For instance, Knöferle and Spence (2012) have reported that stimuli sharing a crossmodal correspondence can induce shorter RTs (reaction times) in a particular task. However, according to the authors, there is little evidence that such stimuli also have perceptual consequences. In line with this hypothesis, Gallace and Spence (2006) have shown that the presentation of either a crossmodally congruent or incongruent sound did not actually change the perceived size of a circle that was presented with it, despite the fact that participants’ RTs changed significantly. Two noticeable exceptions are worth mentioning though. Liang et al. (2013) have shown that rounded shapes enhanced sweetness sensitivity (at least at near-threshold levels), whereas angular shapes did not. Another study has highlighted that low-pitched notes played by brass instruments can enhance the perceived intensity of the bitter taste of caffeine, and high-pitched notes played by the piano can enhance the perceived intensity of sucrose (Crisinel et al., 2012). Thus, some crossmodal correspondences seem to lead to a crossmodal enhancement effect, a phenomenon that is well-known in flavor perception (see Section “Synesthetic Experience”). We will argue in the next section that knowing how to trigger the mechanisms of crossmodal correspondences involved in freshness (if any) could facilitate consumers’ categorization of a given product as fresh or even lead to freshness enhancement. However, if we want to enhance the freshness perceptual experience by triggering specific crossmodal correspondences, it appears necessary to first determine the type of correspondence likely at hand in freshness perception.

### The Crossmodal Perception of Freshness in Beverages

The literature on the sensory contributors to freshness perception can feed hypotheses about the crossmodal correspondences that could exist and potentially lead to a freshness enhancement effect (see Labbe et al., 2009a for a review). Several studies have highlighted that carbonation was part of consumers’ expectations for fresh beverages (e.g., McEwan and Colwill, 1996; Guinard et al., 1998). Moreover, the perception of the carbonation of a beverage may also be influenced by auditory cues provided by the bubbles (Yau and McDaniel, 1992; Guinard and Mazzucchelli, 1996). Zampini and Spence (2005) conducted several experiments to investigate the role of auditory cues in the perception of carbonation in beverages. In a first experiment, the carbonated water samples were judged to be more carbonated when the overall sound level was increased and/or when the high frequency components (2–20 kHz) of the water sound were amplified. They were also evaluated as being more carbonated when they were held close to the ear rather than further away. Another experiment in which the participants had to assess the level of carbonation and the oral irritation of water samples, in the mouth, revealed that neither the perceived carbonation nor the perceived oral irritation were influenced by variations in the level of auditory feedback. Overall, Zampini, and Spence’s results highlight the significant role that auditory cues play in modulating perception of the carbonation of beverages. However, for the perception of carbonation in the mouth, oral-somatosensory and nociceptive cues dominate over auditory cues (see Dessirier et al., 2000; Carstens et al., 2002, for more details on the mode of action of carbonation). Then, a crossmodal correspondence occurring between visual carbonation and the intensity of the trigeminal stimulation due to carbonation can also be expected. Other results have been obtained by recent studies regarding the influence of auditory cues corresponding to the pouring of a beverage on the perception of its temperature (Velasco et al., 2013) or corresponding to the opening of the packaging on the perception of freshness in terms of a new and not-tampered-with product (see Spence and Wang, 2015 for a review). The results obtained by Velasco et al. (2013) have highlighted a crosslevel correspondence between word attributes and sounds that were congruent (“Hot Drink” and the sound of the hot pouring water, and “Cold Drink” and the sound of the cold pouring water).

Regarding flavor, it seems to be difficult to identify semantic ambiguity due to its generic character. Correspondences with a non-perceptual cognitive level (e.g., semantic content) that we characterized as crosslevel correspondences have not been investigated yet in freshness perception. However, in line with the semantically mediated correspondence in which low and high describe stimuli varying in pitch and visual elevation, the semantic ambiguity relative to freshness is more easily identifiable (i.e., multisensory stimulations *versus* aging of fruits and vegetables, see Introduction). It is thus reasonable to suspect potential interactions between the perceptual features themselves that influence freshness perception (e.g., crossmodal correspondences), as well as between the perceptual features and the various meanings assigned to freshness (e.g., crosslevel correspondences).

The perceptual and semantic information is stored in memory and is thus strongly dependent on the participants’ background knowledge. If there is a need to better identify the respective contributions and functions of each sensory modality in the case of freshness perception, it is also important to take into account the influence of the cognitive factors such as attention and memory in studies’ elaboration and analysis of their limitations.

## Memory, Expectations, and Knowledge

### Learned Associations and Expectations

Human beings, at least in western countries, are prone to be in contact with a rich food and drink environment in which several associations between various perceptual features (e.g., colors, texture) can be repeatedly encountered. In the case of a negative post-ingestive effect, people will store this information in memory with respect to the poisonous food they ingested and they will subsequently adapt their dietary behavior. Such past experience will drive long-term flavor preference formation and intake (Myers and Sclafani, 2006).

In the case of freshness perception, Labbe et al. (2009a) have argued that the positive experience of alleviation of unpleasant symptoms (thirst, mouth dryness, mental fatigue, feeling too hot) following consumption of a given beverage leads to a learned association of these positive experiences (e.g., between the coldness of a drink and the relief from the sensation of feeling too hot) with freshness perception. This is in line with what Zellner and Durlach (2002) found among a group of American students. When asked to list foods, beverages, and sensory characteristics they considered to be refreshing, the American students mentioned water most of the time (90% of respondents) as well as cold temperature (see also Eccles et al., 2013). Prior learning may explain other associations with freshness, such as the positive association with clear appearance (Zellner and Durlach, 2003) and the negative associations with sweetness, thickness (McEwan and Colwill, 1996; Guinard et al., 1998; Labbe et al., 2009b), intense flavor, and after-taste (Guinard et al., 1998). Labbe et al. (2009a) concluded that further work is needed before assuming that flavor-refreshing learning is as robust as other types of associative learning such as odorant-sweet taste learning.

Moreover, it should be noted that prior learning can modulate the kinetic aspects of food or drink consumption, contributing to an increase or decrease in the perceived intensity of a particular sensory compound (Blissett et al., 2007). The actual moment of swallowing seems particularly important since it has been shown that this is when major aroma pulses are induced (Buettner et al., 2002; Hodgson et al., 2003), thereby making a major contribution to the final percept of the beverage. However, McCrickerd et al. (2014) have highlighted the notion of product dependence by suggesting that the strength of associations formed between a drink’s sensory characteristics and its post-ingestive effect would be weak compared to that for solid foods. McCrickerd et al. (2014) think that it could potentially be the case because beverages are consumed rapidly, and this reduced oral exposure time may limit the strength of its oro-sensory signal and subsequent learning. Moreover, a high degree of variability in swallowing patterns has been found between individuals, with some individuals performing simple swallowing actions whereas others incorporate learned tasting behaviors into their everyday consumption routines to adapt their consumption habits to their physiological requirements (Blissett et al., 2007; Buettner and Beauchamp, 2010). It is possible to go further, hypothesizing that some people adapt their consumption habits not only at a physiological level, but also to obtain the most pleasant experience, when consuming a drink that they expect to be fresh. Concerning freshness, Westerink and Kozlov (2003) have highlighted that people tend to value temporary sensory input during the actual freshness experience (e.g., temperature), whereas they tend to keep in long-term memory other sensory features related to the freshness percept (e.g., particular compounds such as menthol).

Beyond the perceptual level, we can also wonder how the semantic properties related to freshness perception influence subsequent experiences of freshness. According to Mathis (2002), semantic properties elicit consumers’ knowledge and beliefs (e.g., mental representations built from previous experiences). These mental representations help for stimuli identification, building knowledge of their properties, and the adaptation of consumers’ behavior according to their expectations relative to the product experienced. Regarding the concept of expectations it is worth refining the notion to distinguish two levels of expectations: (i) expectations as perceptual priors, what Seriès and Seitz (2013) named the “structural” expectations resulting from the integration of the various perceptual features of a given stimulus, and (ii) expectations as beliefs (implicit or explicit at a doxastic level, see Dretske, 1988, p. 117 for this distinction) that can be manipulated through instructions, sensory cues, or contextual variables (see Seriès and Seitz, 2013). It should be noted that the generation of different taste/flavor expectations can be a function of the participants’ background and/or culture (Zellner and Durlach, 2003), expertise/experience with particular food or beverage domains (Parr et al., 2003; Smith, 2007), and age (Philipsen et al., 1995).

### Culture and Expertise

The influence of participants’ background and culture has been investigated by Nguyen et al. (2002), who conducted a study highlighting that the odors of vanilla, caramel, strawberry, and mint induced sweetness enhancement in western countries where people often experience those odors with sucrose. By contrast, non-western participants did not describe some of these odors as sweet, probably due to a less frequent pairing of these odors with sweetness in their food culture. In another cross-cultural study (Wan et al., 2014), the same seven drinks were presented in three different types of glass to participants from mainland China, the United States, the United Kingdom, South Korea, and India. The results revealed that the same beverage color sometimes set up distinctly different flavor expectations depending on both the type of receptacle and the cultural background of the participants. These sources of variability relative to background knowledge and culture probably have major influences on the lack of consensus concerning the constitutive properties of freshness in food and beverages (Cardello and Schutz, 2003; Heenan et al., 2008; Zhang et al., 2016).

Regarding the influence of expertise, conflicting results have been reported. A handful of studies have highlighted that the level of expertise can help an individual categorize certain flavor components more easily and may improve perceptual capacities (e.g., the capacity to identify the perceptual similarities between two different products; Ballester et al., 2008). This has been nicely illustrated in the case of wine expertise: based on common mental representations, experts are able to efficiently categorize different odors of two types of wine. It has been argued that this allows them to both organize and use their perceptual knowledge more efficiently (see Hughson and Boakes, 2001 for a review). However, some studies have highlighted that expertise did not necessarily result in enhanced perceptual capacities. For instance, Pangborn et al. (1963) investigated the effect of color on sweetness perception. The addition of color to a solution to give the appearance of a rose wine caused wine experts but not novices to judge the solution as sweeter than colorless controls. We may reasonably hypothesize that level of expertise may impact freshness perception as well, since it has been shown that the color, as well as the sweetness of beverages influence the perceived freshness (e.g., Guinard et al., 1998; Zellner and Durlach, 2003). Moreover, different sensory expectations might be triggered by different perceptual features depending on the consumer’s background knowledge (Zellner and Durlach, 2003).

Another experiment conducted by Morrot et al. (2001) revealed that expertise did not necessarily improve the perceptual capacities of experts. They investigated whether wine experts could consistently associate olfactory descriptors with different types of wines. In the first session, the participants were asked to draw up a list of olfactory descriptors for a white wine and a red wine (based on a list of descriptors that was supplied to them or their own descriptors). Then, the participants had to indicate which of the two wines most intensely presented the character of each descriptor. In the second session, one week later, the same white wine previously presented in session 1 was artificially colored red with an odorless dye and the participants were asked to do the same task of comparison between the red wine and the white wine (colored red). Morrot et al. (2001) results revealed that the white wine artificially colored red in session 2 was described as a red wine in terms of olfactory descriptors by the panel of 54 experts. They suggested the existence of a “perceptual illusion” due to the color change that would influence the pairing between the different wines and their appropriate sensory descriptors.

Regarding freshness, clear color has been reported by consumers to be the most expected color for fresh food or beverages (e.g., Zellner and Durlach, 2002). Thus, it might be that white wines would be considered fresher than red wines. However, it remains an empirical question whether a change in the color of the wines would be sufficient to impact participants’ perception of their relative freshness.

An alternative explanation of Morrot et al. (2001) results has been proposed by Shepherd (2012, p. 139). According to Shepherd, the participants’ attention in Morrot et al.’s (2001) study was biased toward using the same descriptors for what they believed to be the same purpose. However, an important source of inter-individual variability can also partially result from the way the participants attend to a specific task (Stevenson, 2012, see Section “Attention”). The influence of attentional factors on both flavor and freshness is considered in more detail in the next section.

## Attention

Two different modes of attention have been described by Posner (1980): exogenous and endogenous modes of attention. The exogenous mode of attention is underpinned by the salience of certain environmental features, whereas the endogenous mode corresponds to relatively voluntary mechanisms sensitive to learned events. Alleged influences of exogenous, and to a lesser extent endogenous, attentional processes have been suggested for the mouth capture phenomenon occurring in flavor perception. Further investigation is needed to conclude whether the mouth capture phenomenon can be generalized to all instances of flavor such as freshness.

### Exogenous Attentional Processes

Stevenson et al. (2011) manipulated exogenous attention by varying the stimulus characteristics so that either oral or nasal cues became more salient. Their results showed that the participants were prone to shift the localization of the percept toward the physical locus of the more salient cue. Furthermore, Stevenson (2012) has highlighted that the contribution of olfaction to flavor goes largely unnoticed, compared to that of taste and somatosensation, even when participants are asked to detect its presence. Hence, he suggested that exogenous attentional processes may be more important in inducing oral localization than endogenous attentional processes. Therefore, because exogenous attention in turn depends on the stimulus characteristics, we may reasonably expect differences between freshness and other instances of flavor, regarding their subjective location. In the case of beverages, the trigeminal component is likely one of the main contributors to freshness, due to the processing of coldness by the trigeminal cold-sensing neurons for instance (Labbe et al., 2009a). This is not necessarily the case, or at least not to a similar extent, for all types of flavors (e.g., strawberry). We can thus wonder whether the trigeminal component may bias the subjective location of freshness.

Moreover, it has been shown that exogenous attentional processes can also have perceptual consequences regarding the way participants pay attention to the task (e.g., according to the instructions they receive). For instance, while a sweetness enhancement phenomenon can occur after a single co-exposure between a sweet taste and a novel odor, Prescott et al. (2004) have observed that the adoption of a synthetic perceptual strategy during the co-exposure would also be necessary to produce sweetness enhancement. They observed that participants generally used a synthetic perceptual strategy when they were asked to rate the overall flavor intensity of the stimulus. By contrast, when the participants were asked to rate the intensity of several perceptual features of the stimulus separately, they tended to rely more on an analytical perceptual strategy. Sweetness enhancement would thus be dependent on the number and type of rating scales provided to participants. This phenomenon has been called the “halo-dumping” effect; in the case of few rating scales provided, participants tend to dump their ratings for a perceptual feature for which no response scale has been provided (e.g., the intensity of a fruity odor) onto another perceptual feature for which a response scale has been provided (e.g., the sweetness of the fruity odor). However, some studies have provided data that discredit the hypothesis of a systematic halo-dumping effect. For instance, Nguyen et al. (2002) have shown that the odor-taste enhancement effect could occur even when multiple scales are presented.

### Endogenous Attentional Processes

Endogenous attention enables us to extract relevant information relatively early from a rich and complex stimulus environment. That is, stimuli are better processed, in terms of response time and accuracy, when they are anticipated. For instance, the results obtained by Ashkenazi and Marks (2004) have highlighted that endogenous attention can improve the detectability of the gustatory flavorant “sucrose” but not the olfactory flavorant “vanillin”. The authors suggested that olfactory stimuli would be already fully processed before attention is directed toward them, reducing the functional consequence of endogenous attention. When consuming a beverage that consumers expect to be fresh, endogenous attention may be shifted toward a specific locus in the mouth or nose where consumers expect a freshness experience.

To summarize, it clearly appears that the subjective location of a final percept and the respective contributions of each of its sensory contributors is dependent on several parameters that belong to both the perceptual level (e.g., modulating the intensity of some perceptual features) and higher order cognitive levels such as memory and both exogenous and endogenous attentional processes. Starting by investigating the particular influence of crossmodal interactions and correspondences that can occur in freshness perception will allow us to generate new empirical evidence in terms of the multisensory processes at stake. Nevertheless, it is important to bear in mind that these complex multisensory processes are also dependent on conditions that influence the degree of integration. Two major documented conditions are reviewed in the next section.

## Particular Conditions That Influence the Degree of Integration

### Congruency

The term congruency was previously evoked in Section “Synesthetic Experience” to highlight some cognitive processes that occur in flavor representations, giving rise in some cases to crossmodal enhancement phenomena. The existing literature on congruency suggests that several types of congruency can occur at different levels but also that the characterization of congruency is generally vague and even sometimes circular. For instance, Schifferstein and Verlegh (1996) defined congruency as the extent to which two stimuli are appropriate for combination in a food product. According to Shepherd (2012), congruency can be defined as the extent to which two stimuli complement each other. Lim and Johnson (2012) suggested a statistical account of congruency according to which congruency would correspond to the extent to which two stimuli commonly appear together and thus are highly associated in a food. Small and Prescott (2005) suggested a distinction using the term “perceptual congruency” that can be observed, according to the authors, when sniffed odors elicit descriptions of qualities that are more usually associated with basic taste qualities and so may arise as a result of repeated pairing (the terms “contiguity” and “synchrony” are also used by Small and Prescott, 2005). This definition of perceptual congruency is close to that of perceptual similarity used by some researchers when referring to perceptual qualities that are alternatively attributed to different compounds. Lim and Green (2007) conducted several studies based on spatial discrimination between capsaicin and QSO4, which can both induce bitterness and burning sensations on the tongue. They concluded that perceptual similarity is a notion that also refers to functions shared by the different compounds in terms of the perceptual consequences they induce.

Another distinction has been suggested by Spence (2011, p. 972), who argued that crossmodal correspondences may be used by humans “along with spatiotemporal and semantic congruency to help solve the crossmodal binding problem” (i.e., determining which of the many simultaneous afferent stimuli in different modalities should be bound together). The use of “along with” suggests that congruency is something different from, though related to, crossmodal correspondences. According to Spence (2011), perceptual congruency can refer to spatial and/or temporal co-occurrence during multisensory integration, in contrast with another type of congruency, “semantic congruency” that refers to the situations in which pairs of stimuli presented vary in terms of their identity and/or meaning. This definition of semantic congruency has also been used together with alternative terms such as appropriateness and/or compatibility effects between the stimuli (e.g., Piqueras-Fiszman and Spence, 2011) as well as the notion of consistency (see Spence, 2011).

These various ways to define congruency have created some confusion between the very notion of congruency and the proper mechanism of crossmodal correspondences. In particular, it has led some researchers to interpret their results in terms of a certain degree of congruency between pairs of stimuli without considering the cognitive mechanisms at hand. For instance, White and Prescott (2001) concluded that simultaneous orthonasal presentation of “congruent” odors (strawberry) was found to shorten RTs for sweetness taste recognition, relative to “incongruent” odors (grapefruit). However, sweet taste and strawberry odor are often co-experienced from childhood and this association is actually learned through repeated exposure. We suggest that the effect on RTs observed by White and Prescott (2001) seems to result from a typical case of crossmodal correspondence, built from past experience of the regular association of sweet taste and strawberry odor, rather than only congruency between these two perceptual features.

In order to clarify the difference between the phenomenon of crossmodal correspondences and the condition of congruency, two types of congruency that appear to occur at two different levels will be distinguished: (i) the perceptual congruency that refers to the spatial and/or temporal co-occurrence of two or more stimuli during multisensory integration, and (ii) the semantic congruency occurring at a higher cognitive level that helps to determine whether or not two or more stimuli are compatible or consistent in terms of identity and/or meaning.

For instance, in the case of freshness perception in beverages, consistency effects could be assessed by measuring the consequences of presenting the sound of a liquid containing bubbles poured in a glass with either a low or high pitch, and a picture of a glass containing either small or big bubbles. If, as we hypothesize, high-pitched sound is consistent with small bubbles and low-pitched sound is consistent with big bubbles, measurable effects can be obtained such as shorter RTs in consistent blocks, highlighting a positive consistency effect. This type of result can provide evidence of a crossmodal correspondence phenomenon occurring between particular perceptual features that influence freshness perception.

### Unity Assumption

Besides congruency, “the unity assumption” is another condition that the degree of multisensory integration depends on. As for congruency, the very notion of the unity assumption remains vague and ambiguous. Various ways of understanding the unity assumption have been proposed. According to research on this concept (Welch and Warren, 1980; Welch, 1999), the unity assumption corresponds to the observer’s assumption that two or more sensory inputs refer to a single distal event. According to Welch and Warren (1980), the strength of the unity assumption is a function of the number of physical properties (e.g., time, space, temporal patterning, number, shape, size) that are redundantly represented in the stimulus situation, as well as the relative weighting assigned by the observer to these properties. Based on Welch and Warren’s (1980) definition of the unity assumption, Vatakis and Spence (2007) have proposed that whenever two or more sensory inputs are highly consistent in one or more dimension(s) (linked to the physical properties of the stimulus as well as influenced by top–down factors such as the semantic content), observers will be more likely to process them as referring to the same underlying multisensory event rather than as referring to separate unimodal events.

There are at least two ways to characterize the unity assumption: on the one hand, the bottom–up approach includes the physical properties linked to the stimulus, such as spatial and/or temporal co-occurrence which actually overlaps with a certain way of understanding congruency (Spence, 2011). On the other hand, some authors have highlighted that it remains unclear whether the unity assumption refers more to a top–down (i.e., more cognitively mediated) or rather to a bottom–up (i.e., more stimulus-driven) process (see Welch and Warren, 1980; Welch, 1999; Spence, 2007, on this point). The top–down approach to the unity assumption also overlaps with a certain way of understanding congruency since the higher cognitive level of congruency (i.e., semantic congruency) would also serve to solve the crossmodal binding problem (Spence, 2011). It should be noted that it also remains unclear whether the process of unification occurs consciously or unconsciously (Bertelson and de Gelder, 2004; Spence, 2007). Then, two main types of unity assumption will be distinguished: (i) the unity assumption as a bottom–up influence in terms of perceptual priors and (ii) the unity assumption as a top–down influence in terms of beliefs. Both types of unity assumption will influence the integration process and will in some cases lead the observer to consider different afferent sensory inputs to refer to the same multisensory event or object.

As was mentioned earlier, flavor is generally presented as a unified percept resulting from the integration of all senses. This implies that each flavor representation will depend on the unity assumption of the observer concerning the multimodal object experienced. Even though the particular influence of such an assumption has not been empirically explored yet for the perceptions of flavor and freshness, it is reasonable to think that it can modulate the degree of integration in the case of such multisensory perceptions. Considering flavor or freshness as unified percepts or not has various consequences regarding the overall product experienced such as different intensity judgments or different subjective locations of the final percept.

## Conclusion

The present review aimed at exploiting the existing literature on flavor to characterize the perceptual and cognitive mechanisms that underlie the multisensory perception of freshness, in the case of beverages. We have hypothesized that some questions about flavor remained open until now because flavor is a generic term, and in some circumstances its genericity blurs the representational nature of particular instances of flavor such as freshness, as well as the corresponding cognitive mechanisms at hand. In fact, flavor has been characterized in various ways during the past twenty years: it has been suggested that flavor representations could correspond to synesthetic experiences or result from different processes leading to object representations or categories. Given the complexity of the various perceptual and cognitive mechanisms that underpin flavor perception, the main purpose of researchers has been to obtain empirical evidence regarding the respective contributions of each sensory modality. There is a general consensus on the fact that (i) retronasal smell is the main sensory contributor to the experience of flavor even if its implication is generally not consciously perceived, and (ii) the localization of flavor perception has an illusory component due to the mouth capture phenomenon.

Freshness perception has also been described as the result of a multisensory integration and it reasonably corresponds to a particular instance of flavor, underpinned by similar cognitive mechanisms. Similarly to flavor, it appears that freshness perception is characterized by a hybrid content, both perceptual and semantic. In fact, a semantic ambiguity is present regarding freshness since it can alternatively refer to particular sensory stimulations (e.g., coldness, sourness, menthol odor) as well as different characteristics linked to the age or crispness of fruits or vegetables. Due to these different meanings to which freshness can directly refer, it supports a higher degree of specificity compared to flavor. However, the question of the function of flavor objects in the mouth has appeared as a tipping point which has highlighted that considering a particular instance of flavor facilitates the identification of particular functions (e.g., the alleviation of mouth dryness in the case of freshness). Moreover, it is important to bear in mind that freshness potentially differs from flavor with respect to (i) the sensory modality that constitutes its main contributor, (ii) its subjective location (e.g., mouth capture in the case of flavor), and (iii) the typology of its modulating factors.

In order to characterize the perceptual and cognitive mechanisms that underlie the experience of freshness, we focused on the case of crossmodal interactions and correspondences (see Section “Crossmodal Interactions and Correspondences”) which can induce measurable effects such as shorter RTs (e.g., faster product categorization) and crossmodal enhancement phenomena resulting from the interaction between two or more sensory inputs under certain circumstances. A distinction has been introduced between the crossmodal correspondences that can occur between two sensory modalities at a perceptual level and the correspondences that involve higher order cognitive levels, in the case of interactions between perceptual and semantic features that we characterized as crosslevel correspondences. This is of particular interest from an applied perspective since freshness features are part of the consumers’ sensory expectations and likely determine food and beverage acceptance and appreciation. Indeed, knowing how to trigger the mechanisms of crossmodal or crosslevel correspondences regarding freshness could facilitate consumers’ categorization of a given product as being fresh or even lead to freshness enhancement. However, crossmodal correspondence mechanisms still remain to be explored in freshness perception. To enhance the freshness perceptual experience, it is important (i) to identify the specific perceptual features contributing to freshness perception and to specify their respective weights, according to the stimulus context considered (e.g., beverages), and (ii) to obtain empirical evidence of the different types of correspondences that occur regarding freshness perception.

This approach must also consider the impact of top–down influences such as memory, expectations, and background knowledge on freshness, similarly to what has been thoroughly investigated in flavor perception (see Sections “Memory, Expectations, and Knowledge” and “Attention”). For instance, regarding freshness in the case of beverages, the positive learned associations following a particular drink consumption will be stored in memory and will influence the subsequent experiences. The multisensory processes potentially leading to crossmodal enhancement or crossmodal correspondences are function of particular conditions such as the different forms of congruency. Two different types of congruency have been distinguished in this review: on the one hand, the perceptual congruency that refers to the spatial and/or temporal co-occurrence between two or more stimuli during multisensory integration and on the other hand the semantic congruency occurring at a higher cognitive level that helps to determine whether or not two or more stimuli are consistent in terms of identity and/or meaning. The concept of the unity assumption, which has been defined as the condition under which different afferent sensory inputs are processed as referring to the same multisensory event or object, has been analyzed. The fact that there are two ways of interpreting the unity assumption in the literature has been underlined: on the one hand the unity assumption as a bottom–up influence in terms of perceptual priors and on the other hand, the unity assumption as a top–down influence in terms of beliefs.

**FIGURE 2:**
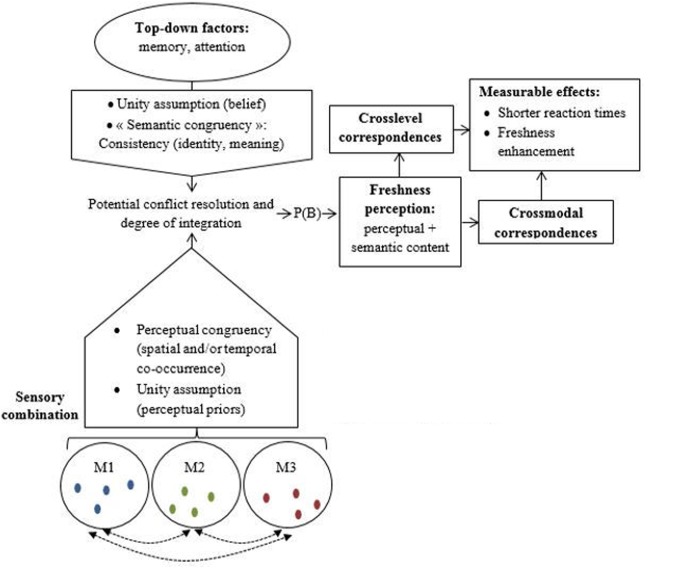
Schematic sequences of the multisensory integration processes leading to freshness perception. M1; M2; M3: Sensory modalities; 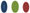 Perceptual features; 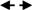 Crossmodal interactions; P(B): Binding probability.

Although the majority of these particular multisensory processes have been reported in flavor perception, they still remain to be investigated regarding particular instances of flavor such as freshness. From our analyses and the conceptual distinctions that have been introduced, we propose a model of freshness perception that will pave the way for further empirical research in the food and beverage domain, and more precisely on flavor and freshness perception (**Figure 2**).

## Author Contributions

JR, JL, and MA contributed to the theoretical elaboration, organization of the structure of the arguments, reviewing of the full article. JR and JL wrote most parts of the article.

## Conflict of Interest Statement

The authors declare that the research was conducted in the absence of any commercial or financial relationships that could be construed as a potential conflict of interest.

## Footnotes

1 In the present review, we use “cognitive mechanisms” or “cognitive factors” to refer to the set of mechanisms involved in the extraction and integration of information that constrains, influences, and interacts with the everyday experience of food and drink (Verhagen and Engelen, 2006).

2 In addition to the crossmodal enhancement that can result from the sensory fusion between a conscious taste and an unconscious smell, another kind of crossmodal enhancement can also be obtained for instance when one odor and one taste at subthreshold are presented together; Dalton et al. (2000) have shown that the threshold for detecting the odor was then lowered and the participants were sometimes able to detect the odor even if the individual perceptual features were weak.
